# Cardiac Magnetic Resonance Imaging in Pulmonary Arterial Hypertension: Ready for Clinical Practice and Guidelines?

**DOI:** 10.1007/s11897-020-00479-7

**Published:** 2020-09-01

**Authors:** Barbro Kjellström, Anthony Lindholm, Ellen Ostenfeld

**Affiliations:** 1 Department of Clinical Sciences Lund, Clinical Physiology and Skåne University Hospital, Lund University, SE-221 85 Lund, Sweden; 2 Swedish Pulmonary Arterial Hypertension Registry, Uppsala Clinical Research Centre, Uppsala University, Uppsala, Sweden

**Keywords:** Pulmonary arterial hypertension, Ventricular remodelling, Atrial remodelling, Pulmonary artery, Tissue characterization, Outcome, Risk assessment

## Abstract

**Purpose of Review:**

Pulmonary arterial hypertension (PAH) is a progressive disease with high mortality. A greater understanding of the physiology and function of the cardiovascular system in PAH will help improve survival. This review covers the latest advances within cardiovascular magnetic resonance imaging (CMR) regarding diagnosis, evaluation of treatment, and prognostication of patients with PAH.

**Recent Findings:**

New CMR measures that have been proven relevant in PAH include measures of ventricular and atrial volumes and function, tissue characterization, pulmonary artery velocities, and arterio-ventricular coupling.

**Summary:**

CMR markers carry prognostic information relevant for clinical care such as treatment response and thereby can affect survival. Future research should investigate if CMR, as a non-invasive method, can improve existing measures or even provide new and better measures in the diagnosis, evaluation of treatment, and determination of prognosis of PAH.

## Introduction

**Fig. 1:**
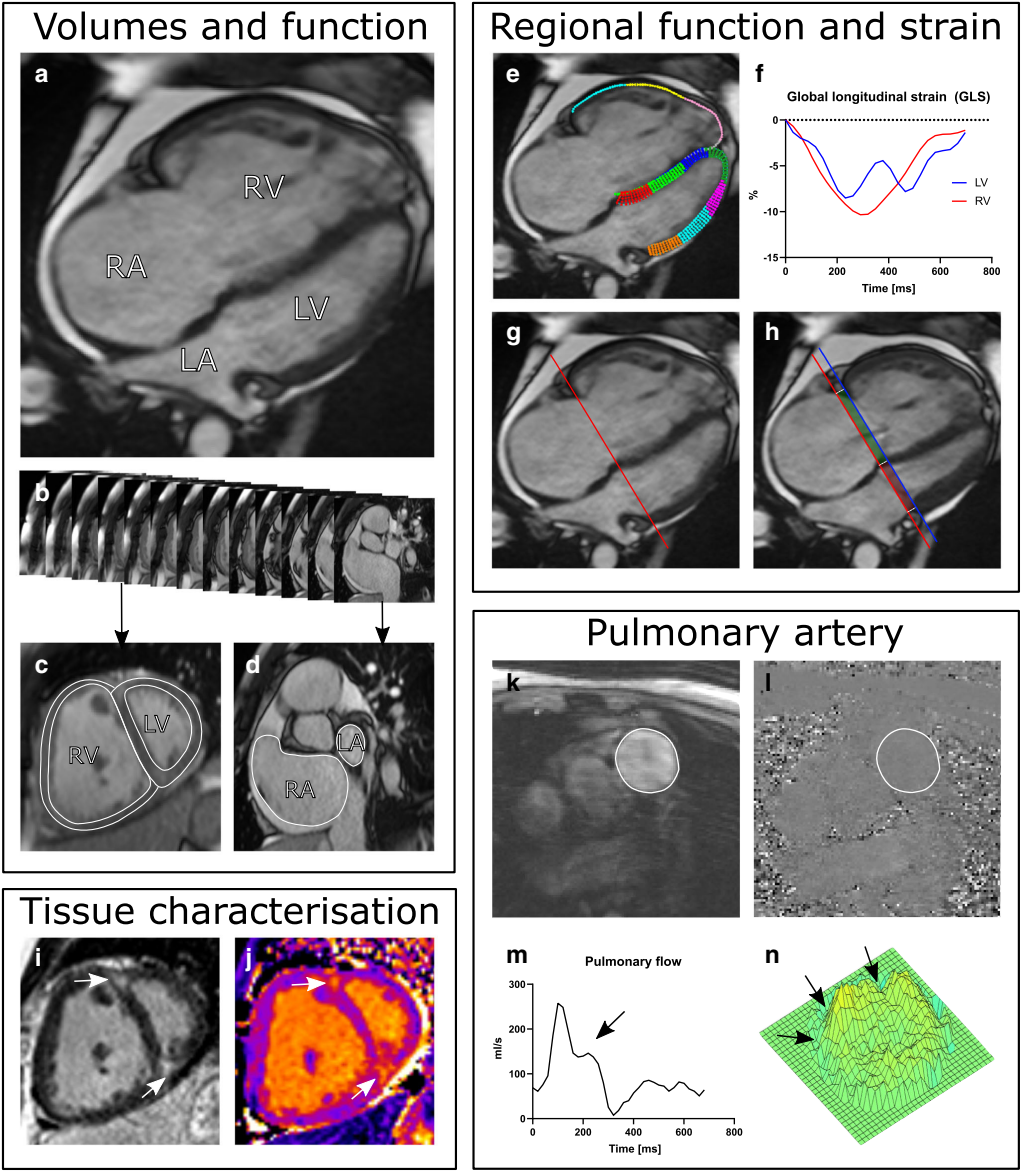
Cardiovascular magnetic resonance (CMR) images in a patient with idiopathic pulmonary arterial hypertension. (A) Cine image of 4-chamber view in end diastole showing an enlarged right ventricle (RV) and atrium (RA) and a small left ventricle (LV) and atrium (LA). The RV is hypertrophied, and pericardial effusion is present. (B) Cine short axis stack covering the heart from apex to the base is used for the volumetric assessment of ventricle (C) and atria (D). (C) Example of epicardial and endocardial delineations of both ventricles (in white) and (D) endocardial delineations of both atria. (E) RV and LV tracking for strain analysis in 4-chamber view. (F) Time resolved strain analysis curves for RV and LV (here global longitudinal strain (GLS)). (G) Atrio-ventricular plane in end diastole (red line) in 4-chamber view and (H) in end systole (blue line). Atrio-ventricular displacement (AVPD) is measured as the distance moving from base to apex between the red line in end diastole and the blue line in end systole. The longitudinal contribution to stroke volume (SV) is the volume encompassed by the atrio-ventricular plane marked with blue colour in the left ventricle and green colour in the right ventricle. (I) Phase-sensitive inversion recovery late gadolinium image of short-axis view showing RV insertion fibrosis (white arrows) and (J) increased native T1 values in the corresponding areas. (K) Anatomical view of the pulmonary artery delineated in white. (L) Phase-contrast imaging of the pulmonary artery delineated in white from which the flow is computed. (M) Time-resolved pulmonary flow curve during one cardiac cycle. Notice the systolic notch (black arrow), which is indicative of increased pulmonary vascular resistance [111, 120, 121]. (N) 3D plot of pulmonary flow marking the velocity of each voxel from late systolic phase. Simultaneously with the systolic forward flow, backward flow (arrows) is present in the posterior part of the pulmonary artery. This patient had the following data: *Volumes and function*—RV: end-diastolic volume 356 ml, end-systolic volume 284 ml, stroke volume 72 ml, ejection fraction 20%, mass 83 g; LV: end-diastolic volume 117 ml, end-systolic volume 72 ml, stroke volume 45 ml, ejection fraction 39%, mass 88 g; RA maximum volume 292 ml, LA maximum volume 51 ml. *Strain and regional function*—peak LV GLS − 8.5%, peak RV free wall GLS − 9.2%, RV atrio-ventricular plane displacement 11.2 mm, RV longitudinal contribution to SV 64%, RV lateral contribution to SV 36%, LV atrio-ventricular plane displacement 7.5 mm, LV longitudinal contribution to SV 53%, LV lateral contribution to SV 43%, septal contribution to SV 5%. *Tissue characterization*—T1 values 1420 ms (increased) at the RV insertion points and 1030 ms (normal) in the RV and LV. *Pulmonary artery*—pulmonary net flow 66 ml, peak velocity 52 cm/s, mean velocity 17 cm/s, area 15.03 cm^2^, distensibility 0.13%/mmHg

Pulmonary arterial hypertension (PAH) is a progressive disease with increased vascular resistance and arterial pressure in the pulmonary circulation. Symptoms such as dyspnoea and fatigue are vague, while there can be a long latency and delay to diagnosis [1]. Mortality is high and most commonly related directly or indirectly to right ventricular (RV) function [1]. While echocardiography currently is the first-line modality to assess cardiac function, assessment of RV volumes and function is challenged by the one- and two-dimensional nature of echocardiography [2–5]. With the complexity of the RV structure, cardiovascular magnetic resonance imaging (CMR) plays an important role in the diagnosis and follow-up of patients with PAH [1, 6]. CMR is the gold standard for cardiac volumes, function, blood flow, and mass (Fig. 1), due to its high accuracy and reproducibility. Furthermore, CMR offers tissue characterization of the ventricular myocardium. In the 2015 ESC/ERS guidelines for diagnosis and treatment of pulmonary hypertension, the only imaging-related parameters included in the risk stratification are right atrial area and pericardial effusion with evidence from echocardiography alone (Table 1) [1]. However, several CMR-related parameters are proven relevant for diagnosis, assessment of disease severity, and prognostication. The purpose of this review is to provide an overview of the latest advances within CMR regarding diagnosis, evaluation of treatment, and prognostication of patients with PAH.

**Table 1:**
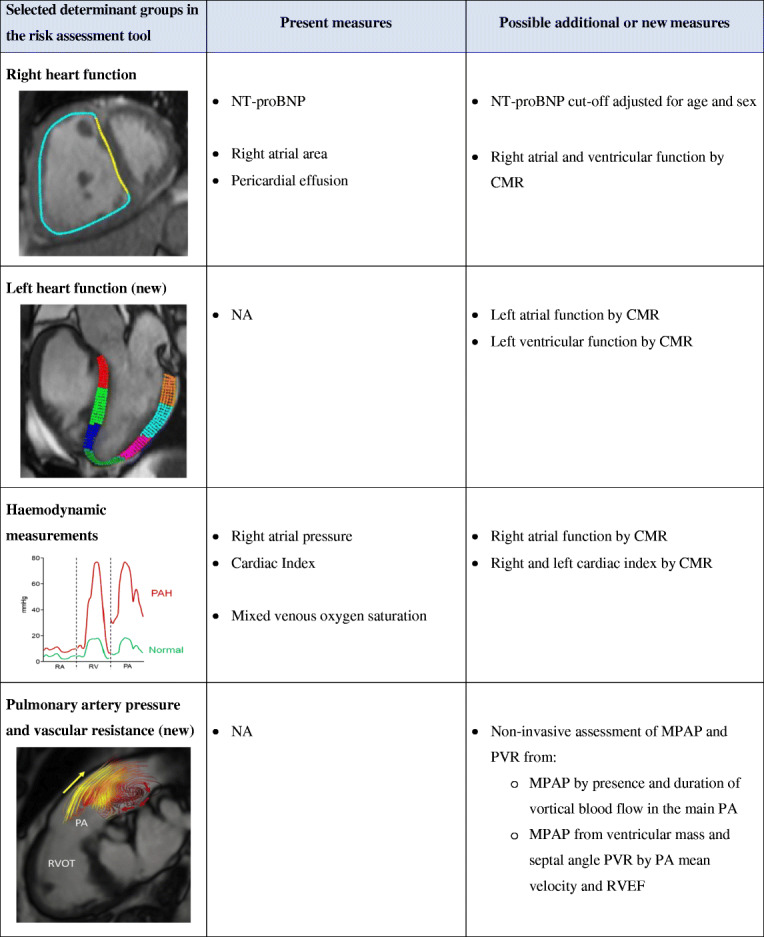
Determinant groups and measures for risk assessment and suggestion for possible adjustments to current measures and/or addition of new measures [1, 115–117]

In the table, the column to the right suggests new measures that could be additional, alternative, or a replacement of measures included in the current ESC/ERS risk stratification (middle column). In line with the scope of this paper, focus is put on how non-invasive measures with CMR might add to the risk assessment in PAH. The CMR images illustrate some of the possible variables of right and left heart function and pulmonary arterial measures that could be considered included in guideline-recommended risk stratification. Here illustrated with an example of right ventricular circumferential strain of the free wall (magenta) and septum (yellow) at a midventricular level, left ventricular longitudinal strain in a three-chamber view (each colour represents a segment), and pulmonary artery vortex formation with posterior retrograde flow (red arrows) during systolic forward flow (yellow arrows) (reprinted from [119], with permission from Elsevier)

*WHO* World Health Organization, *6MWD* 6-min walked distance, *NT-proBNP* N-terminal pro-brain natriuretic peptide, *CMR* cardiovascular magnetic resonance imaging, *NA* not applicable, *MPAP* mean pulmonary artery pressure, *PVR* pulmonary vascular resistance, *PA* pulmonary artery, *RVEF* right ventricular ejection fraction, *RVOT* right ventricular outflow tract

## Mass, Volumes, and Function

The increased afterload in PAH can lead to a compensatory RV hypertrophy (Fig. 1(A)), increased ventricular mass index (defined as RV mass divided by left ventricular (LV) mass), and increased RV and atrial volumes (Fig. 1(B–D)).

### Mass

The ventricular mass index has been associated with outcomes such as all-cause death in patients with Eisenmenger PAH [7] and with the composite endpoint comprising a combination of all-cause and cardiopulmonary death, lung transplant, rehospitalization, and clinical worsening in patients with PAH [8–10]. However, in a meta-analysis from 2016, RV mass and ventricular mass index were not predictive of all-cause death in PAH [11]. This finding was confirmed in a more recent systematic review and meta-analysis, where RV mass was only related to composite endpoint and not all-cause death [12••]. On the contrary, a compensatory RV hypertrophy has been linked to a better survival, while a decrement in RV mass at serial examinations was a sign of poor prognosis. Both of these results indicate that adaptive RV modelling is beneficial for patients with PAH [13].

### Ventricular Volumes and Ejection Fraction

Increased RV volume (Fig. 1(C)) and reduced LV volume, RV ejection fraction (RVEF), and stroke volume (SV) are noted to be prognostic markers for PAH [14]. When used in conjunction with existing risk assessment tools, these CMR markers show increased value over current prognostic methods for risk classification [15, 16••]. After adjusting for age, sex, and body surface area, 11% could be reclassified as having a higher risk and 36% a lower risk of 1-year mortality [16••].

RVEF has been shown to be the strongest predictor of mortality among these variables in patients with pulmonary hypertension [11, 12••]. When including only patients with pulmonary *arterial* hypertension, RVEF was the only parameter predicting death and the composite of adverse events comprising all-cause and cardiopulmonary mortality, rehospitalization, lung/lung-heart transplant, and clinical worsening [12••]. It is worth noticing that when excluding patients with congenital heart disease from the PAH group, RVEF only predicted adverse events, not mortality [12••]. This is in agreement with three recent studies on patients with non-congenital PAH, in which RVEF was not associated with death or lung transplant [17•, 18, 19].

### Atrial Volumes and Function

The importance of right atrial volume (Fig. 1(D)) and function is becoming more clear regarding the prognosis of patients with PAH [9, 17•, 20, 21••, 22, 23], as they have been shown to be associated with clinical worsening [9]. Patients with right atrial maximum volumes > 74 ml/m^2^ doubled their risk for death or lung transplant compared with patients with normal right atrial volumes [17•]. Moreover, reduced left atrial volumes could be indicative of a LV underfilling in PAH [17•] and have been presented as an indicator of poor prognosis [24, 25].

## Regional Function and Strain

Ejection fraction is a crude measurement and while many patients with PAH have a preserved LVEF, and at times even a preserved RVEF, this should not be mistaken for a normal ventricular function. Regional RV function, such as myocardial strain (a deformation measured as a change in length; ∆*L*/*L*), RV fractional area change, and tricuspid annular plane systolic excursion (TAPSE), are regularly assessed in patients with PAH using echocardiography [1, 2]. Novel techniques to characterize regional ventricular function with CMR are emerging [20, 23, 26–31]. However, and of note, echocardiographic and CMR equivalent measures are not directly interchangeable [32].

### Myocardial Strain

It is well documented that RV and atrial strain measured by CMR are lower in patients with PAH than in controls [20, 26–29]. LV global longitudinal strain is also lower, despite preserved LVEF (Fig. 1(E, F)) [21••, 27, 29]. Therefore, in addition of being a diagnostic tool, myocardial strain might have utility in prognosis and follow-up of treatment response in patients with PAH [20, 21••, 28]. Reduced strain increases the risk for adverse events such as death, lung transplant, and functional class deterioration, incurring a hazard ratio (HR) of 1.06 for LV longitudinal strain, 2.52 for RV longitudinal strain, and even as much as 4.5 for RV circumferential strain [28]. Importantly, LV and RV longitudinal strain values increased after initiation of PAH-dedicated treatment [21••]. The improved strain values correlate with improvements in known prognostic markers of PAH such as 6-min walk test, pro-BNP, and mean pulmonary arterial pressure (MPAP) [21••]. Furthermore, an interventricular dyssynchronous contraction has been documented with a left-to-right delay assessed by strain in adult and paediatric patients with PAH [33–36].

As such, strain assessment with CMR is increasingly interesting in the evaluation of patients with PAH. Moreover, it shows that there are left-sided implications of this otherwise considered right-sided disease. The implications are important for understanding the pump physiology and mechanisms of the disease as well as for finding early signs of treatment effect.

### Atrio-Ventricular Plane Displacement

Atrio-ventricular plane displacement (and tricuspid annular plane systolic excursion), regional contribution to SV, and RV fractional area change are novel CMR techniques for evaluating regional cardiac function in PAH [23, 30, 31, 37]. Both tricuspid annular plane systolic excursion and fraction area change are shown to have a good correlation with invasive measures such as pulmonary vascular resistance (PVR) index, MPAP, and RV stroke volume index [18]. In addition, tricuspid annular plane systolic excursion ≤ 18 mm, RV fraction shortening ≤ 16.7%, and RV fractional area change ≤ 18.8% are associated with survival in PAH, with HRs of 4.8, 3.6, and 3.8, respectively.

Right atrio-ventricular plane displacement (Fig. 1(G, H)) has been shown to be lower in patients with pulmonary hypertension compared with controls, while the longitudinal contribution to RV stroke volume did not differ between the groups, owing to increased RV diameter and lower SV among patients [31]. Interestingly, LV atrio-ventricular plane displacement and longitudinal contribution to LV SV were both lower in patients with PAH than controls, despite a preserved LVEF in both groups [31]. However, the importance of these regional alterations regarding morbidity and mortality is unknown.

## Tissue Characterization

Myocardial tissue characterization in PAH has been suggested as a prognostic marker using late gadolinium enhancement (LGE) and T1 values [1] (Fig. 1(I, J)).

### Late Gadolinium Enhancement

A gadolinium-based contrast is distributed in relation to the amount of extracellular space. This results in an increased concentration of gadolinium and consequently higher signal intensity (hyperenhancement) in myocardial scar, fibrosis, or infarction compared to viable myocardium. Typically for PAH, hyperenhancement is present at the RV insertion (Fig. 1(I)) [8] and associated with poor clinical status and survival [8, 38–41]. However, if the fibrosis stretches into the interventricular septum, hyperenhancement appears to have a stronger association with outcome than fibrosis in the RV insertion alone [38].

The appearance of fibrosis can vary among aetiologies of PAH. As such, in patients with congenital heart disease and PAH (e.g. Eisenmenger with right-to-left shunt and PAH), the fibrosis in the right ventricle and septum does not fully resemble that of non-congenital PAH, as in idiopathic PAH or PAH associated with connective tissue disorders, such as scleroderma [38, 42]. Furthermore, patients with sclerodermas have been shown to have intrinsic myocardial involvement besides PAH-related alterations [29, 43–48]. In addition, localized LV fibrosis and infarctions have been shown even in cardiac asymptomatic patients with scleroderma suggesting a more complex fibrosis pattern in this population of PAH [43, 44].

### T1 Mapping

While localized fibrosis can be detected by LGE, diffuse pathology such as general myocardial inflammation and diffuse fibrosis can be assessed using T1 mapping. Furthermore, T1 mapping can be performed both before and after contrast administration which enable calculation of extracellular volume fraction (ECV) in the myocardium (Fig. 1(J)) [43, 49–56]. T1 mapping and ECV for tissue characterization are relatively new features applied to patients with PAH [57–65] and have been shown elevated which indicate fibrosis in the RV insertion points [57, 59–61, 63, 66].

It is, however, still unclear how T1 values should be interpreted, as studies are diverging on whether values are higher in patients with PAH compared with controls [57, 61, 63, 67] or not [58–60, 66, 68]. It should be noted that there is a multitude of different sequences for T1 mapping of which some are heart rate dependent [69]. Patients with PAH are prone to having high heart rate. This is a concern, if special measures are not taken in the acquisition of images [69]. Furthermore, as many of the studies comprise diverse groups of patients, pooling the values into a uniform conclusive value could be considered infeasible. As an example, patients with scleroderma, including those cardiac asymptomatic, have been shown to have increased T1 values [44]. However, the distribution can stand in contrast to patients with congenital heart disease with fibrosis in the right ventricle and septum that does not resemble that of non-congenital PAH [42]. The diversity of patients as well as different sequences are drawbacks for finding a generalizable cut-off value for pathology and outcome. Moreover, the low spatial resolution (1.8 mm × 1.8 mm × 8–10 mm) when assessing the relatively thin RV wall (on average 3–5 mm) is a caveat for generating reliable values as the risk of accidentally including blood in the trabeculations or epicardial fat in the assessment is substantial [49, 50, 69]. One should therefore interpret the T1 values of the right ventricle with caution.

## Estimates of Mean Pulmonary Pressure and Pulmonary Resistance

Cardiac and pulmonary artery (PA) pressures and resistance are essential parts of both diagnosis and prognosis of PAH (Fig. 1(K–M)) [1]. Current guidelines denote MPAP ≥ 25 mmHg (> 20 mmHg is borderline pulmonary hypertension), PVR ≥ 3 WU, and a normal function of the left ventricle (PA wedge pressure (PAWP)) ≤ 15 mmHg as manifesting the diagnosis [1, 70]. These key measures determine treatment response [1, 70] and are recommended to be obtained from invasive right heart catheterization (RHC) [1]. While RHC incurs a low morbidity and mortality rate, there are still risks of complications during the intervention, and the examination includes radiation exposure [71]. However, non-invasive methods are emerging as promising alternatives [72, 73].

### Estimation of Pulmonary Arterial Pressure with CMR

Estimation of MPAP with CMR using ventricular mass index and interventricular septal angle [74], including RV function along with PA size (Fig. 1(K, L)) [75], has been performed in patients with pulmonary hypertension and chronic obstructive pulmonary disease and showed moderate to good correlation with RHC-derived MPAP. While each of these measurements seems plausible, the use of multiple variable calculations for estimations of values introduces possible sources of error, which is why more direct measures are preferable.

RV pressure overload results in an interventricular shift of the septum toward the left ventricle in patients with PAH (Fig. 1(C)) [76–78]. Leftward septal bowing occurs when the RV pressure is ≥ 5 mmHg higher than LV pressure [79]. This will contribute to an altered filling of the left ventricle—i.e. cause an underfilling and a decreased LV stroke volume [34]. The ventricular septal curvature has been shown to correlate with systolic PA pressure with a premise of the close correlation between RV systolic pressure and systolic PA pressure [79]. Quantification of the septal curvature duration index (defined as the proportion of CMR frames with a septal bow toward the left that was present during one cardiac cycle) has been shown to be associated with worse prognosis if it lasts > 2/3 of the cardiac cycle [80].

A more recent, and direct, method for estimating MPAP by CMR assesses the presence and duration of vortical blood flow in the main PA (Table 1) [81••, 82–85]. A vortex is a formation of concentric ring- or spiral-shaped curves [86–89] and is an effect of coexisting forward flow and retrograde flow at the posterior wall during systole in the main PA (Fig. 1(N)) [81••, 82–85]. The premises for estimating MPAP from vortex formation are (1) detection of a vortex (indicating increased resistance and decreased elastance) and (2) the time it exists in relation to the full cardiac phase (to evaluate the pressure increase). With these prerequisites, vortex duration has been shown to be accurate in the identification of pulmonary hypertension (a vortex duration ≥ 14.3% of the cardiac cycle resulted in sensitivity of 0.97 and specificity of 0.96 of detecting pulmonary hypertension) [85]. The accuracy of MPAP needs, however, to be verified in larger, prospective studies from other groups. Furthermore, investigation of the possible effects of pulse wave reflection and PA trunk width would, in the context of vortex formation in the PA, be of interest.

### Estimation of Pulmonary Vascular Resistance and Stiffness with CMR

PVR, assessed by RHC, is calculated as a ratio of the mean pressure gradient and blood flow in the PA [1]. Non-invasive PVR has been suggested using CMR PA flow metrics (Fig. 1(M, N)) (average and peak velocities) [90–94]. In a meta-analysis, a multitude of different methods (some a combination of PA and RV variables) were compiled and showed a high correlation with PVR from RHC (pooled *r* = 0.81 (95% CI 0.74, 0.87)) [95]. Combining RV measures with PA flow metrics (Fig. 1(M)) adds important components in cases with advanced stages of PAH and high PVR, when the PA does not distend further. Hence, the average PA velocity in this late state will only reduce slightly, while RVEF will be more affected [92].

PA stiffness occurs before severe symptoms develop and is an early manifestation of PA remodelling [96–99]. Thus, direct measurements of the stiffness might add accuracy and value to the diagnosis and prognostication of PAH [96, 97]. PA distensibility is one of several measures of PA stiffness and reflects the degree of vascular remodelling as the percent increase in pulmonary vessel diameter in relation to the increase in pressure [74, 96, 100]. It is a strong prognostic marker [74] and has been associated with RV pulmonary arterial uncoupling in patients with unexplained exercise intolerance and normal resting echocardiography results [101].

A novel measure reflecting PA stiffness is PA velocity transfer function, which describes the influence of vessel geometry and compliance/stiffness causing frequency-dependent changes in the input velocity profile (in the proximal part of the PA) as it travels through the artery and thus produces an output velocity profile (in the distal part of the PA) [102•]. PA velocity transfer function is strongly associated with invasive measures of PA impedance, stiffness, and vascular resistance. Furthermore, changes in PA velocity transfer function have been shown to be independent of elevation in PAWP. This could be perceived as an advantage in the aging PAH population, as PAWP is affected by age and comorbidities [103]. However, it is yet unknown if alterations in PA velocity transfer function are related to morbidity and outcome.

## Arterial-Ventricular Coupling

RV failure occurs when the right ventricle can no longer adapt to the elevated pulmonary vascular load. RV pulmonary arterial coupling refers to the energy transfer between ventricular contractility and arterial afterload. It reflects the load imposed upon the right ventricle, as a measure of the right ventricle compensation to the increasing PA stiffness [104–107]. Ventricular contractility is a load-independent measure of systolic function and can be expressed as end-systolic elastance (Ees) [108, 109•, 110•]. Arterial afterload is the net vascular stiffness and can be expressed as arterial elastance (Ea) [108]. RV pulmonary arterial coupling, measured as Ees/Ea (end-systolic elastance/arterial elastance), has been presented as being useful for prognostication in PAH [111]—to detect pending RV failure [112] in patients with preserved RVEF [109•], for example.

Simultaneous information on both function and loading conditions can be interpreted from RV pressure-volume loops. These are in general generated from invasive measures from RHC and volumes. Computation of RV pressure-volume loops could, besides assessing Ees, Ea, and Ees/E, be of interest in the investigation of stroke work, potential energy, and ventricular efficiency [107]. A non-invasive computation of pressure-volume loops has been shown to be applicable on the left side using a time-varying elastance model, CMR, and brachial pressure [113]. However, calculating potential energy and mechanical efficiency on the right side requires RV pressure values and an estimation of the RV volume at zero pressure, the *V*_0_. Both linear regression models [106, 107] and a fixed value [104, 105] have been used to determine *V*_0_, and future studies are needed for a fully non-invasive computation of RV stroke work and ventricular efficiency as applicable in pulmonary hypertension.

## Outcome and Risk Assessment

Risk stratification to predict outcome in PAH is vital in the individualization of treatment strategies and improvement of survival (Table 1). Several tools, of different complexity, have been developed [114–118]. To be accepted in daily practice, the tool needs to be clinically applicable and simple to use. On the other hand, PAH is a complex disease and requires advanced investigation to detect disease progression [1] and thus, for a risk assessment tool to be useful, oversimplifying could be a mistake.

Right atrial measures, such as pressure, volume, or area, are not part of the diagnosis, but are important prognostic parameters that can be obtained with RHC, echocardiography, or CMR [1, 2, 119, 120]. It should be noted that in the ESC/ERS risk stratification, the only imaging variables currently included are the right atrial area and pericardial effusion and no RHC measure [1]. In the REVEAL risk score, pericardial effusion is the only imaging-related parameter, while RHC measures of MPAP and PVR are also included [114].

Despite improved treatments and treatment strategies, survival for patients with PAH is still poor. At the first 1-year follow-up after diagnosis, only 17–29% of patients were in a low risk according to the ESC/ERS risk stratification tool [115–117]. There are several ways to construe this information, but two plausible interpretations are that either treatment is not effective enough yet or that other variables need to be assessed for a better prediction—or a combination of these.

## Conclusion

Pulmonary arterial hypertension is a progressive disease with high mortality. Haemodynamic measurements, utilizing right heart catheterization, are the gold standard for diagnosis in PAH and to some extent for prognosis. To date, substantial effort is put into mimicking these measures using non-invasive methods like echocardiography and CMR. However, several CMR markers carry prognostic information in themselves and incur a better survival when improved. It is thus warranted that future research investigates these non-invasive methods to see if they can improve existing measures or even provide new and better measures in the diagnosis, evaluation of treatment effect, and determination of prognosis of PAH.
